# Recent fermentation developments for improved rhamnolipid production

**DOI:** 10.1007/s00253-025-13517-y

**Published:** 2025-06-13

**Authors:** Phavit Wongsirichot, James Winterburn

**Affiliations:** Department of Chemical Engineering, The University of Manchester, Oxford Road, Manchester, M13 9PL UK

**Keywords:** Rhamnolipids, Biosurfactants, Fermentation, Process development, High productivity

## Abstract

Rhamnolipids (RL) are microbial amphiphilic molecules containing a hydrophilic rhamnose head and a hydrophobic fatty acid tail. RL are of interest to academia and industry due to their potential as a biosurfactant, being used to substitute petroleum-based surfactants in traditional applications or in novel bioremediation and biomedical applications. Currently, commercialization of RL is still in a nascent state, and improved RL production in terms of titers, yields, and productivities could benefit their techno-economic viability and market competitiveness. This review provides a detailed assessment of recent studies that have achieved higher RL production through improvements in microbial producers, media formulation, fermentation design, and operations. Key successes and areas for future work are identified and discussed in detail, as well as put into context with pilot-scale and techno-economic analysis of RL production from the wider literature. This review provides an updated perspective on the current status of RL production. The discussions and insights provided could potentially be used to improve future RL and biosurfactant commercialization efforts.

## Introduction

**Fig. 1:**
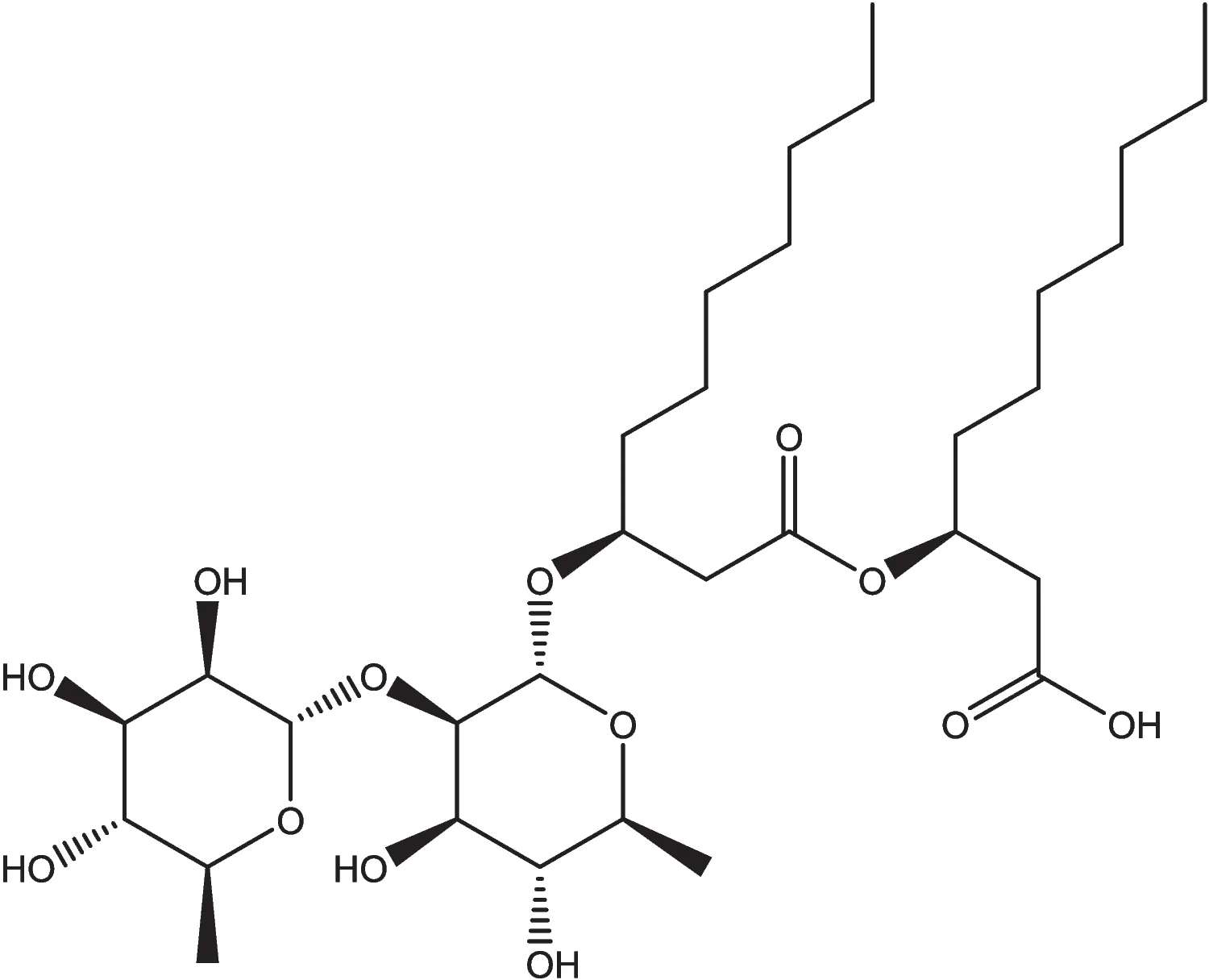
An example rhamnolipid structure (2-O-α-l-rhamnosyl-α-l-rhamnosyl-3-hydroxydecanoyl-3-hydroxydecanoic acid)

Rhamnolipids (RL) are a family of biosurfactants of significant interest within academia and industry. RL are produced from microorganisms, such as various *Pseudomonas* species (Guzmán et al. 2024). The amphiphilic RL molecule consists of a hydrophilic head and a hydrophobic tail, with the head being composed of one to two rhamnose molecules, while the tail is made up of saturated or unsaturated β-hydroxy fatty acid chains (typically of 8–24 carbons) (Guzmán et al. 2024). There are a wide variety of RL congeners with over 60 identified (Abdel-Mawgoud et al. 2010; Jiang et al. 2020). An example structure of an RL is seen in Fig. 1.

RL have promising traditional surfactant-related properties, such as wetting, foaming, and emulsifying properties, as well as differentiating properties that make them novel surfactants, such as biodegradability and antimicrobial activity. Thus, RL have potential roles in many traditional and novel applications, including cleaning products, cosmetics, and personal care products; agricultural green pesticides; bioremediation; oil recovery; and biomedical applications such as immunomodulation, anti-tumor treatment, and wound healing (Banat et al. 2000; Chen et al. 2017; Guzmán et al. 2024; Zhao et al. 2022). In particular, RL could provide advantages over traditional petroleum-derived surfactants through their lower toxicity, biodegradability, and sustainable production routes (Banat et al. 2000; Guzmán et al. 2024), leading to their exploration as potential candidates to penetrate the over 40 billion USD global surfactant market (Markets and Markets 2023). Recently, the first commercial RL production plant was opened by Evonik in Slovakia, with 1150 m^3^ of total fermentation volume (Evonik Industries AG 2021; Evonik Industries AG 2023; Monagas 2022). Nevertheless, commercialization of RL and biosurfactants in general is still hindered by cost-competitive disadvantages, including low yields and high substrate and operating costs (Moutinho et al. 2021). RL production can be impacted by a variety of fermentation-related factors including producer organism and metabolic regulation, substrate choice and concentrations (including sources of carbon, nitrogen, phosphorus, iron, and sulfur), temperature, pH, dissolved oxygen, degree of foaming, and shear stresses (Blunt et al. 2022). This, in turn, means that a myriad of strategies can be utilized to improve RL production.

This review is designed to provide an in-depth and critical assessment of the recent developments for improved RL production within the literature (from 2020 to 2024). The significance of this review stems not only from the updated picture of innovations within the field but also from its scope and approach compared to the wider RL review literature, as this review places at its core the strategies utilized by recent studies to improve RL titer, productivity, and/or yield, as well as providing context with respect to process scale-up and process techno-economics. Potential gaps that could be improved in future studies are also elucidated. This review is designed to complement other existing reviews, such as the earlier review by Blunt et al. (2022) on RL production. In particular, the in-depth focus on improving RL production allows it to stand out from, as well as complement, the broader and more generalized reviews within the wider RL literature, such as Moutinho et al. (2021) and Guzmán et al. (2024). Due to the significant differences in RL productivity, solid-state fermentation is not included in this review, as further development is still needed for it to compete with submerged fermentation in RL production. The information, discussions, context, and recommendations provided by this review should allow it to act as a jumping-off point for future RL research, development, and commercialization efforts.

## Recent developments in rhamnolipid production

**Fig. 2:**
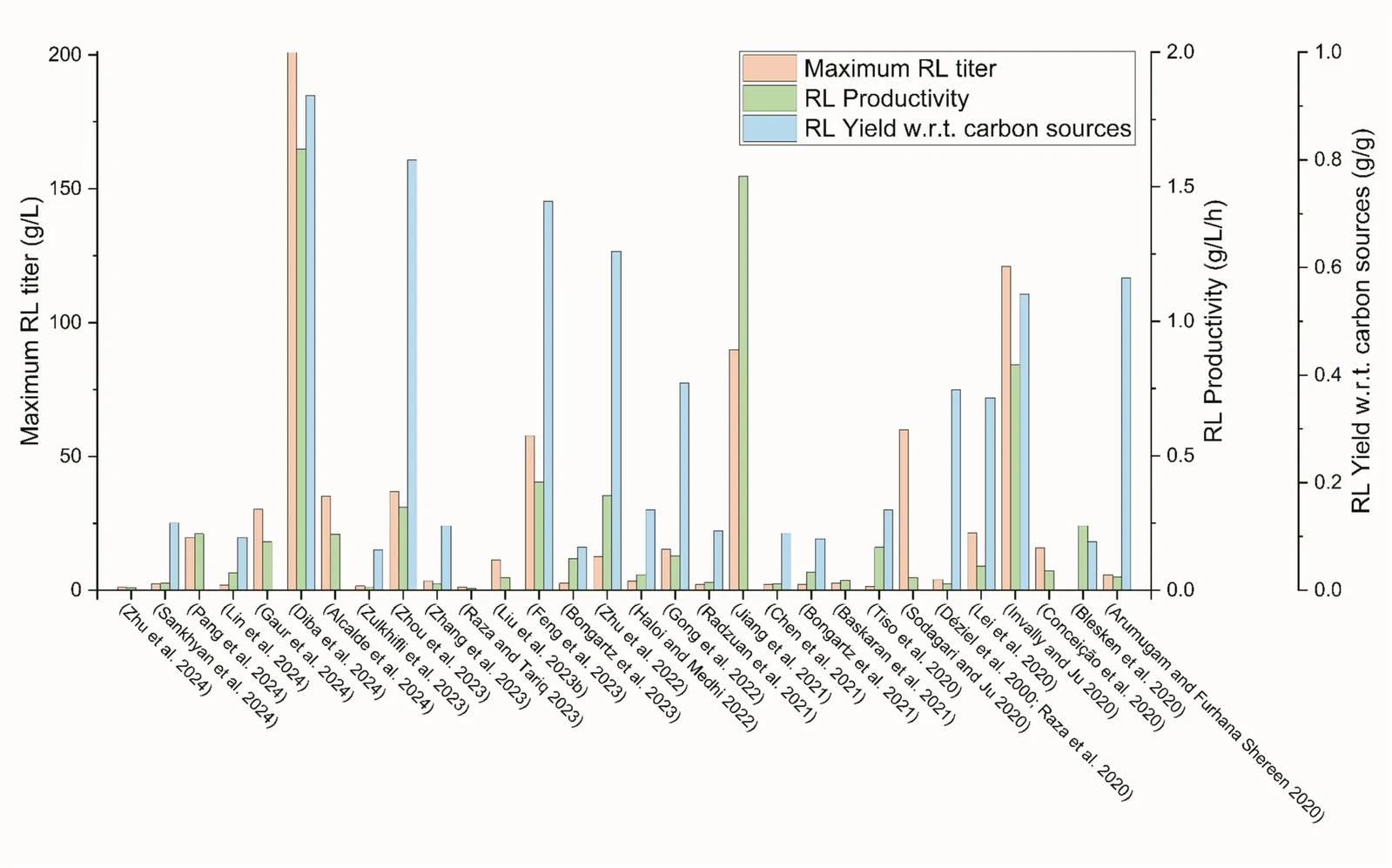
Rhamnolipid titer, productivity, and yield achieved by recent studies with notable developments in rhamnolipid production approaches

A summary of recent studies with notable developments in rhamnolipid production is provided in Table 1, with the relevant RL production performance data. Figure 2 illustrates the wide range of RL titers, productivities, and yields with respect to carbon source reported by these studies. Not all of the studies referenced provide these RL production performance indicators directly or, in some cases, provide insufficient data to calculate these important fermentation metrics. To improve the usability of rhamnolipid production literature for scale-up and commercialization, future studies should provide and discuss all these key metrics with respect to the fermentation aspect investigated.

**Table 1:** Summary of recent studies with notable developments in rhamnolipid production

Notable aspects of the study	Producer organism	C-source(s)	N-source(s)	Fermentation setup (working/total volume)	Maximum dry cell weight	Maximum RL titer	Overall RL productivity	Overall RL yield w.r.t. carbon sources^a^	Reference
Novel producer, media optimization, process optimization	*Pseudomonas aeruginosa *GB-3	Glucose	Ammonium sulfate	Batch shake flask (100 mL/-)	-	1.06 g/L	0.00883^b^ g/L/h	-	Zhu et al. (2024)
Media formulation, media optimization	*Pseudomonas aeruginosa* NG4	Glucose (1% w/v) and crude glycerol (1% w/v)	Ammonium molybdate tetrahydrate (0.39 g/L)	Batch shake flask (0.4–1.2 L/1–5 L)	-	2.2–2.5 g/L	0.0229–0.0260^b^ g/L/h	0.11–0.125^b^ g/g	Sankhyan et al. (2024)
Novel producer, media formulation, media optimization	*Pseudomonas putida* AB-BDAC-Δflag	Glucose (N/a) and crude glycerol (20 g/L with feeding)	Yeast extract (30 g/L) and ferric ammonium citrate (0.3 g/L)	Fed-batch bioreactor (2 L/5 L)	-	19.77 g/L	0.210 g/L/h	-	Pang et al. (2024)
Fermentation setup, media formulation	Oil field–isolated *Pseudomonas aeruginosa*	Soybean oil or diesel (20 g/L)	Yeast extract (2 g/L) and ammonium sulfate (1 g/L)	Batch shake flask bubble fermentation (500 mL/500 mL)	-	1.97 g/L	0.0656^b^ g/L/h	0.0985^b^ g/g	Lin et al. (2024)
Process optimization, fermentation setup	*Pseudomonas aeruginosa* gi |KP 163922|	Waste engine oil (controlled at 5% v/v)	Ammonium sulfate (1 g/L with feeding)	Fed-batch bioreactor (2 L/3 L)	9.23 g/L	30.22 g/L	0.180^b^g/L/h	-	Gaur et al. (2024)
Fermentation setup, media formulation, process optimization	*Pseudomonas aeruginosa* HK02	Sunflower and soya oil (40 g/L with feeding)	Sodium nitrate (8 g/L with feeding)	Continuous bioreactor (1 L/5 L)	16 g/L	201 g/L	1.64 g/L/h	0.92 g/g	Diba et al. (2024)
Media formulation, media optimization	*Pseudomonas aeruginosa* 6 K-11	Corn oil (70 g/L)	Sodium nitrate (for 21.172 C:N ratio)	Batch shake flask (100 mL/500 mL)	~ 12 g/L	35.124 g/L	0.209^b^ g/L/h	-	Alcalde et al. (2024)
Fermentation setup	*Pseudomonas aeruginosa* USM-AR2	Waste cooking oil (2% v/v)	Sodium nitrate (5.5 g/L)	Batch fluidized bed reactor (100 mL/-)	-	1.58 g/L	0.013 g/L/h	0.0754^b^ g/g	Zulkhifli et al. (2023)
Novel producer, fermentation setup	*Pseudomonas aeruginosa* KT1115 Δ*paeKI*Δ*fhp*Δ*nirB*	Rapeseed oil (60 g/L)	Yeast extract (3–6 g/L) with sodium nitrate feeding (0.85 g/L)	Two-stage fed-batch bioreactor (3 L/5 L)	~ 22.5 g/L	36.97 g/L	0.308 g/L/h	0.8 g/g	Zhou et al. (2023)
Fermentation setup	*Pseudomonas aeruginosa* BC1	Glycerol (30 g/L)	Yeast extract (0.01 g/L) and sodium nitrate (2 g/L)	Batch bioreactor with membrane separation (7 L/10 L)	-	3.6 g/L	0.0257^b^ g/L/h	0.12^b^ g/g	Zhang et al. (2023)
Media formulation, salinity stress	*Pseudomonas aeruginosa* EBN-8	Wasted soybean oil (2% w/v)	Carbamide (0.2 g/L)	Batch shake flask	3.99 g/L	1.24 g/L	0.00738^b^ g/L/h	-	Raza and Tariq (2023)
Fermentation setup, media formulation, media optimization, process optimization	*Pseudomonas aeruginosa* D1	Waste frying oil (20% w/v)	Sodium nitrate (6 g/L)	Batch bioreactor with cell immobilization and in situ foam fractionation (2 L/3 L)	-	11.29 g/L	0.0470^b^ g/L/h	-	Liu et al. (2023b)
Novel producer	*Pseudomonas aeruginosa* WJPAB	Rapeseed oil (80 g/L)	Sodium nitrate (6 g/L)	Batch shake flask (40 mL/250 mL)	-	57.83 g/L	0.402^b^ g/L/h	0.723^b^ g/g	Feng et al. (2023)
Novel producer, Fermentation setup, process optimization	*Pseudomonas putida KT2440* SK4	Glucose (10 g/L with feeding)	Ammonium sulfate (2 g/L with feeding)	Fed-batch bioreactor with novel agitation (2 L/3.3 L)	6.34 g/L	2.59 g/L	0.118 g/L/h	0.08 g/g	Bongartz et al. (2023)
Novel producer	*Acinetobacter calcoaceticus* BU-03	Glucose (20 g/L)	Peptone (10 g/L)	Batch Bioreactor (1.4 L/3 L)	3.4 g/L	12.7 g/L	0.352 g/L/h	0.63 g/g	Zhu et al. (2022)
Process optimization	*Pseudomonas *sp. TMB2	Glucose (~ 30 g/L)	Sodium nitrate	Batch beaker with in situ foam fractionation (-/2 L)	~ 7.5 g/L	~ 3.5 g/L	0.0583^b^ g/L/h	0.15 g/g	Haloi and Medhi (2022)
Novel producer, process optimization	*Pseudomonas aeruginosa* E7	Soybean oil (40 g/L)	Sodium nitrate (5 g/L)	Batch bioreactor (4 L/7.5 L)	10.3 g/L	15.4 g/L	0.128^b^ g/L/h	0.385^b^ g/g	Gong et al. (2022)
Media formulation	*Pseudomonas aeruginosa* PAO1	Palm fatty acid methyl ester (20 g/L)	Sodium nitrate (1 g/L)	Batch bioreactor (1.5 L/2 L)	2.09 g/L	2.11 g/L	0.03 g/L/h	0.11 g/g	Radzuan et al. (2021)
Fermentation setup	*Pseudomonas aeruginosa* PAO1	Corn oil (4.5–7.5%)	Sodium nitrate (8–24 g/L)	Sequential fed-batch fermentation Shake flask (60 mL/250 mL)	~ 30–40 g/L	64.4–89.8 g/L	1.54 g/L/h	-	Jiang et al. (2021)
Fermentation setup, novel producer	UV mutated *Pseudomonas aeruginosa*	Kitchen waste oil (controlled at 10 g/L via pulse feeding)	Yeast extract (5 g/L) and ammonium sulfate (1 g/L)	Continuous bioreactor with in situ foam fractionation (5 L/10 L)	-	2.206 g/L	0.024 g/L/h	0.106 g/g	Chen et al. (2021)
Fermentation setup, process optimization	*Pseudomonas putida KT2440* SK4	Glucose (13 g/L with feeding)	Not stated	Fed-batch bioreactor with novel aeration (2.1 L/3 L)	8.7 g/L	2.23 g/L	0.067 g/L/h	0.096 g/g	Bongartz et al. (2021)
Media formulation, media optimization, process optimization	*Pseudomonas aeruginosa* RS6	Treated waste glycerol (1% v/v)	Sodium nitrate (0.2 M)	Batch shake flask (50 mL/250 mL)	-	2.73 g/L	0.0379^b^ g/L/h	-	Baskaran et al. (2021)
Novel producer, media formulation, fermentation setup	*Pseudomonas putida KT2440* *Δflag SK4*	Glucose (10 g/L)	Delft medium	Batch bioreactor with in situ foam fractionation (1 L/1.3 L)	2.8 g/L	1.5 g/L	0.16 g/L/h	0.15^b^ g/g	Tiso et al. (2020)
Media formulation	*Pseudomonas aeruginosa* E0340	Soybean oil (pulse feeding)	Ammonium chloride, yeast extract, and peptone (pulse feeding)	Fed-batch bioreactor with novel aeration (1 L/3 L)	~ 15 g/L	~ 60 g/L	0.048^b^ g/L/h	-	Sodagari and Ju (2020)
Fermentation setup, media formulation, process optimization	*Pseudomonas putida* 300-B mutant	Waste soybean oil (10 g/L) and glucose (1 g/L)	Sodium nitrate (2 g/L)	Batch shake flask	2.95 g/L	4.1 g/L	0.0244 g/L/h	0.373^b^ g/g	Déziel et al. (2000); Raza et al. (2020)
Novel producer	*Pseudomonas aeruginosa* SG ΔpslAB ΔphaC1DC2	Glycerol (60 g/L)	Sodium nitrate (4.87 g/L)	Batch	-	21.5 g/L	0.0896^b^ g/L/h	0.358^b^ g/g	Lei et al. (2020)
Process optimization	*Pseudomonas aeruginosa* E0340	Soybean oil (100 g/L with feeding of 20 g/L per day)	Ammonium chloride (5.76 g/L), yeast extract (5 g/L), peptone (5 g/L), and ammonium nitrate (pulse feed)	Fed-batch bioreactor (1 L/3 L)	30 g/L	120.9 g/L	0.839 g/L/h	0.550^b^ g/g	Invally and Ju (2020)
Process optimization, Media optimization	*Pseudomonas aeruginosa* PAO1	Glycerol (6% v/v) and corn bran water extract (1:1 v/v to medium)	Ammonium sulfate (1 g/L)	Batch shake flask (3 mL/125 mL)	-	15.8 g/L	0.0731^b^ g/L/h	-	Conceição et al. (2020)
Novel producer, fermentation setup	*Pseudomonas putida* KT2440 GR20_RL	Glucose (20 g/L with feeding)	Ammonium sulfate (2 g/L) with ammonium hydroxide pH control	Batch bioreactor with in situ foam fractionation (2 L/1.3 L)	~ 10 g/L	-	0.24 g/L/h	0.09 g/g	Blesken et al. (2020)
Media formulation, media optimization, process optimization	*Enterobacter* *aerogenes* MTCC 8558	*Calophyllum inophyllum* oilcake (10 g/L)	Urea (C:N ratio of 5:1)	Batch bioreactor (1 L/2 L)	-	5.81 g/L	0.0484^b^ g/L/h	0.581^b^ g/g	Arumugam and Furhana Shereen (2020)

^a^Not including additional carbon compounds unaccounted for in the media

^b^Calculated using data from the referenced literature

There are several aspects of the RL fermentation process that can be addressed to improve RL production. In this section, the discussion centers on broad groupings of improvements to (1) the producer organism, (2) media, and (3) fermentation setup. It should be noted that many works have assessed the improvement of multiple aspects in tandem (Table 1) and may be discussed across multiple sections. These more multifaceted approaches to improving RL production could be relevant to RL commercialization by assessing how multiple strategies can work together and if there are any synergies or conflicts between approaches.

### Novel rhamnolipid producers

Numerous recent studies listed in Table 1 have investigated the use of novel RL producers to improve production. There are studies in the wider literature that have reported the isolation of new strains, such as the isolation of *Pseudomonas aeruginosa* from oil and oil-contaminated soil (Zhu et al. 2024), or the use of different microorganisms such as *Acinetobacter calcoaceticus*, which provides advantages over the workhorse *Pseudomonas aeruginosa* as it is non-pathogenic (Zhu et al. 2022). While reasonable RL production can be achieved using these novel strains, such as RL titers and yields of 12.7 g/L and 0.63 g/g carbon source using *Acinetobacter calcoaceticus* (Zhu et al. 2022), only studies where the RL production performance of novel producers was compared to control cultures of typical RL producer(s) will be discussed in detail. This is to negate the impact on performance data that could be caused by different fermentation setups. As a result, the studies discussed are mainly those that involve modification of known RL producers, namely *Pseudomonas aeruginosa* and *Pseudomonas putida*, which were compared to the unmodified wild-type or less modified strains. These results elucidate the biological aspect of the producers that can be changed to improve the overall RL production, e.g., the carbon flux through desirable metabolic pathways.

As *Pseudomonas aeruginosa* is the workhorse of RL production, it is not surprising that recent studies have sought to further improve on existing producer strains. For example, Feng et al. (2023) replaced the original promoter of the rhlAB gene with a stronger PoprL promoter. The rhlAB gene encodes for the RhlAB rhamnosyltransferase I complex, where the subunits, RhlA and RhlB, act as key enzymes during RL synthesis, with RhlA being responsible for the conversion of R-hydroxydecanonyl-CoA to 3-hydroxydecanonyl-3-hydroxydecanonate and RhlB being responsible for the conversion of 3-hydroxydecanonyl-3-hydroxydecanonate to mono-rhamnolipid (Feng et al. 2023). Thus, a stronger promoter for the rhlAB gene should lead to more RL. Indeed, the genetically engineered strain WJPAB was able to achieve higher RL titers in shake flasks, at 57.83 g/L, 91.17% higher than the wild strain WJ (Feng et al. 2023). Notably, high RL productivity and yield were also achieved, at 0.402 g/L/h and 0.723 g/g, respectively, making their strain a promising candidate for use with more sophisticated fermentation regimes.

Aside from enhancing RL metabolic pathways, inhibiting the production of other metabolites is also a viable avenue to improving the overall RL production if carbon flux can be redirected towards RL. Lei et al. (2020) produced single knockout *P. aeruginosa* strains, SG ΔpslAB and SG ΔphaC1DC2, with inhibited production of Psl exopolysaccharides and polyhydroxyalkanoate biopolymer, respectively. This resulted in increased RL production of 21% and 25.3% for SG ΔpslAB and SG ΔphaC1DC2 strains, respectively. Furthermore, combining both changes in a double mutant strain, SG ΔpslAB ΔphaC1DC2, led to an even greater increase in RL produced of 69.7%, to 21.5 g/L (Lei et al. 2020).

Addressing other metabolites can also play a role in RL production, as demonstrated by Zhou et al. (2023) on the potential link between the nitrate metabolism pathways and RL production, particularly on the role of nitric oxide as a signaling molecule in RL production. They found that intracellular nitric oxide concentration is linked to RL production through the development of the strain KT1115ΔpaeKIΔnirS, engineered from *P. aeruginosa* KT1115, which resulted in an 87% reduction in nitric oxide accumulation and a 93% reduction in RL production. Here, the removed nirS gene, corresponds to NirS nitrite reductase, an important enzyme for converting nitrite to nitric oxide. They then demonstrated that improving intracellular nitric oxide accumulation by changing the nitrogen flux through the nitric oxide cycle can improve RL production. Firstly, the engineered strain KT1115ΔpaeKI was used for further modification instead of KT1115 due to higher transformation efficiency. Afterwards, the fhp gene was removed to remove NO dioxygenase, which converts the intracellular nitric oxide to nitrate. The nirB gene was also removed to remove nitrite reductase, NirBD, which converts nitrite to ammonia, thus directing the nitrite to nitric oxide via the aforementioned NirS nitrite reductase. The resulting KT1115ΔpaeKIΔfhpΔnirB strain could accumulate more nitric oxide via nitrate metabolism compared to the KT1115ΔpaeKI and had 2.3 times the RL productivity, at 0.25 g/L/h. A feeding strategy for sodium nitrate was later utilized to further improve RL production (discussed later in the “Fermentation process design and optimization” section). Another study that confirms the importance of nitrogen for RL production is Sodagari and Ju (2020), who found that prolonged nitrogen starvation led to RL production ceasing, impacting the maximum fermentation run time and maximum RL produced.

*P. aeruginosa* can also be modified to indirectly improve RL production. For example, Gong et al. (2022) found that optimization of pH could reduce the high foaming behavior known to inhibit RL production (discussed later in the “Fermentation process design and optimization” section). While non-foaming RL fermentation could be achieved at pH 5.5, this resulted in reduced RL titer. However, ultraviolet and ethyl methanesulfonate compound mutagenesis could be used to improve RL titer from 8.1 to 15.4 g/L, which the authors associated with changes in metabolic flux and an increase in the types of rhamnolipid homologs. Although it should be noted that while the RL titer was improved, it remained lower than the 22 g/L RL titer obtained by the original strain in the shake flask fermentation at normal conditions. Therefore, while the combination of acidic pH and producer modification could lead to improved non-foaming RL production, further improvements are still needed to make this scheme competitive with the control. Further bioprocess development for these novel *P. aeruginosa* strains will also be needed, in order to assess how much additional improvement to RL production can be achieved when they are grown in fed-batch bioreactors with tailored conditions.

Aside from *P. aeruginosa*, the non-pathogenic *Pseudomonas putida* has also been modified for improved RL production. *P. putida* is a chassis of interest due to its non-pathogenicity, rapid growth, and ability to use a wide variety of carbon and nitrogen sources (Tiso et al. 2016; Wigneswaran et al. 2016). For example, the RL synthesis pathway can be introduced to *Pseudomonas putida* KT2440. Pang et al. (2024) demonstrated a significant RL titer of 19.77 g/L and productivity of 0.210 g/L/h, which the authors state to be the best RL production performance to date using metabolically engineered *P. putida* KT2440 (Pang et al. 2024). In this study, the authors not only introduced RL synthesis pathway but also deleted genes corresponding to competitive pathways, used endogenous and synthetic promoters to fine-tune rhlAB expression and enhance the supply of the RL precursor dTDP-l-rhamnose by heterologous rmlBDAC expression.

Another recent example of improved RL production in *P. putida* was conducted by Tiso et al. (2020). First, a comparison was made between different types of gene editing for RL production, using *P. putida* KT2440 containing plasmid pPS05 (Psyn16) or integrons P14ffgrhlAB (strain SK4) and PnagAarhlAB (strain SK40). As strain SK4 was the best performing for both RL and precursor (3-(3-hydroxyalkanoyloxy)alkanoate) production, it was used for further modifications. The authors also improved RL production by chassis optimization, i.e., decreasing the metabolic and energetic burden of the cell. This was conducted by removing genes associated with the flagellar machinery as well as polyhydroxyalkanoate biopolymer synthesis, the latter being a similar approach to the aforementioned Lei et al. (2020) on *P. aeruginosa*. The flagellum knockout strain achieved a 130% higher RL titer than *P. putida* SK4. Meanwhile, different knockout approaches were used with respect to the polyhydroxyalkanoate synthesis pathway. Knocking out the gene phaG, which encodes PhaG, a 3-hydroxyacyl-acyl carrier protein-coenzyme A transferase in the PHA synthesis pathway, resulted in a 27% increase in RL titer, while removing the PHA operon (phaC1ZC2DFI) resulted in a 115% increase in RL titer. This could be the upper limit as a double knockout strain (removing both phaG and phaC1ZC2DFI) did not further improve RL titer. It would have been interesting to have a complementary analysis of the carbon flux, to determine if this is indeed the limit of carbon directed to RL by inhibiting PHA synthesis. It should also be noted that despite the improvements, the RL titer is still relatively low, at around 1 g/L, with low RL yields of around 0.10 g/g. This is in contrast to the higher performance by Pang et al. (2024) with their modified *P. putida*. The modified strain from Tiso et al. (2020) was later used for a novel fermentation setup by Bongartz et al. (2023) (discussed later in the “Fermentation process design and optimization” section), although with a similar order of magnitude of RL production.

Finally, the recent example of *P. putida* conducted by Blesken et al. (2020) should be discussed. Like Gong et al. (2022), the modification was not directly done to improve metabolic flux, but rather to improve the fermentation under certain conditions, in this case to improve in situ foam fractionation performance. Several strains were edited to remove genes for flagellum, fimbriae, specific surface proteins, and exopolysaccharides production, in order to reduce the accumulation of bacteria at the gas–liquid interface to reduce bioreactor cell loss. It was found that through modifications, the biomass content of the foam was reduced by 46%. The cumulative deletion-strain *P. putida* KT2440 GR20_RL was the most efficient producer with an RL productivity of 0.24 g/L/h; in comparison, the SK4 strain had only 0.17 g/L/h (Blesken et al. 2020). This strain had a cumulative deletion of genes related to lapA and lapF, pyoverdine, flagellum, alginate, cellulose, and exopolysaccharide a and b. Although the comparison to strains with deleted genes for synthesis of exopolysaccharide a and b, alginate, and fimbriae and pili seems to suggest that these structures had no significant impact on bacterial foam adhesion, therefore, they may not be necessary for improved in situ foam fractionation.

Due to the complexity of genetic engineering, many studies often only focus on a particular aspect of the genome. More ambitious examples, such as Zhou et al. (2023), Tiso et al. (2020), and Blesken et al. (2020) with multiple modifications, should be emulated in the future. While such an attempt could lead to reduced stability of the cells/chassis, achieving a stable strain that can combine many of the improvements, notably the reduction of carbon flux towards exopolysaccharides, biopolymers, and certain cell features, could be a crucial step in creating the ideal workhorse for the RL industry. This is especially crucial since the productivity of RL commercial regimes will need to compete not only with traditional surfactants but also with other biosurfactants such as sophorolipids, for which higher productivities have been demonstrated (Wongsirichot et al. 2021). Again, it is also important to note that many of these studies exploring novel strains are conducted in sub-optimal fermentation conditions and further bioprocess development and the use of more sophisticated fermentation schemes, such as fed-batch or integrated separation and fermentation, should be conducted for these novel producers, in order to properly quantify the maximum levels of RL titers, productivities, and yields which are achievable at bench scale. This, in turn, will be an important foundation for further scale-up and commercialization, covered in the “Recent development in the context of scale-up and techno-economics” section.

### Media formulation and optimization

#### Media substrate choices

The next aspect of RL production to be addressed is the fermentation media. In the context of this review, improvements to media formulation are the change of certain substrates within the media to replace the substrates typically used within the literature. While media optimization is the optimization of the substrate concentrations or proportions with respect to RL production, preferably via statistical optimization techniques such as surface response methodology. Media formulation and optimization are key areas for the bioprocess development needed for RL producers mentioned in the “Novel rhamnolipid producers” section.

In this section, the studies that investigated the use of media formulation, media optimization, or both, to achieve improved RL titers, productivity, or yields are discussed. With respect to media formulation, many studies compare the use of various substrates and their impact on RL production, often in combination with a rudimentary single-factor assessment of the impact of their concentration within the media. Tiso et al. (2020), discussed in the “Novel rhamnolipid producers” section, compared four different minimal media, to select the most suitable for their *P. putida* KT2440 SK4, selecting Delft medium. However, as seen from the relatively low RL titer of 1.5 g/L, bespoke media for RL production is still likely warranted for commercial RL schemes.

Several studies explored the impact of different types of lipid and oil-related carbon feedstocks on RL production. For example, Lin et al. (2024) explored media improvements for a novel setup using immobilized *P. aeruginosa* with alginate capsules for RL production. Immobilization itself will contribute to improved RL titers. For example, RL titers for free *P. aeruginosa* were relatively low, at approximately 0.6 to 0.4 g/L (Lin et al. 2024). Immobilization using vacant calcium alginate resulted in much higher titers of approximately 1.5 g/L, likely due to improved mass transfer. This demonstrates the potential of the use of immobilized cells in bioremediation coupled with RL production, as well as the potential for setups on substrate utilization. Unfortunately, a direct comparison of free bacteria on diesel without alginate capsules was not provided. Additionally, assessment using a wider variety of lipid and oil-related carbon feedstocks would be beneficial. The specific impact of various immobilization approaches is discussed further in the “Fermentation process design and optimization” section. Diba et al. (2024) assessed a wider range of lipid-related carbon sources, comparing sunflower, paraffin, transgenic soya, olive, *Nigella sativa*, and a mix of sunflower and soya oil for RL production. Although the RL titer was not directly measured, the mix of sunflower and soya oil was found to be the best carbon source based on achieving the lowest critical micelle concentration. Meanwhile, a 10% carbon source was found to provide the highest cell growth (Diba et al. 2024).

Meanwhile, Radzuan et al. (2021) explored the use of novel palm oil refinery by-products as feedstocks, namely palm fatty acid distillate (PFAD) and fatty acid methyl ester (FAME) for RL production. RL titers were 1.06 and 2.1 g/L for PFAD and FAME, respectively. However, no optimization or comparison to more conventional feedstocks was made. Therefore, it is likely that the maximum RL production potential has not been reached for these feedstocks. The authors did state that the theoretical RL production using the Monod equation is an order of magnitude higher than the experimental result (Radzuan et al. 2021). This illustrates the importance of combining improvements to media formulation and optimization of media composition and beyond, to feeding regimes and rates.

Baskaran et al. (2021) also looked at by-products as feedstocks, utilizing waste glycerol from biodiesel production that had undergone treatment, including impurity removal and sulfuric acid treatment, as well as untreated waste glycerol. Technical grade glycerol was used as a control. RL titers of 2.73, 2.64, and 1.95 g/L were achieved for treated waste glycerol, untreated waste glycerol, and technical grade glycerol, respectively. The higher RL from the treated glycerol was likely due to the presence of free fatty acids, fatty acid methyl esters, and other micronutrients. Additionally, cell growth (optical density) in the treated waste glycerol was doubled compared to the untreated waste glycerol. This importantly demonstrates the potential that feedstock pretreatment could have on media components, substrate, and inhibitor content and ultimately RL production. Baskaran et al. (2021) also looked at the impact of the nitrogen source including sodium nitrate, potassium nitrate, ammonium nitrate, and ammonium chloride, with sodium and potassium nitrate producing approximately 1.8 g/L of RL, while ammonium nitrate and ammonium chloride achieved around 1.6 g/L of RL (Baskaran et al. 2021). Therefore, the choice of carbon source does appear to have more impact than the nitrogen source, likely due to the nitrogen source not being directly involved in RL production. As aforementioned, nitrogen levels can impact RL production, as nitric oxide acts as a signaling molecule in RL production (Zhou et al. 2023). Another study that compared different nitrogen sources was Raza and Tariq (2023), who utilized sodium nitrate, carbamide, and ammonium chloride at varying concentrations of wasted soybean oil. Up to approximately, 0.95, 1.02, and 0.75 g/L RL titers were achieved using 0.2 g/L of sodium nitrate, carbamide, and ammonium chloride, respectively. Again, the degree of improvements due to changes in nitrogen source is significant but does appear to be low compared to changes in carbon source. Additional improvements in RL production were achieved by inducing salinity stress using 9 g/L of NaCl, with the RL reaching up to 1.24 g/L.

Returning to selecting media carbon sources, similar to Diba et al. (2024), other recent studies have also found that mixed carbon sources in the media lead to improved RL production. Sankhyan et al. (2024) showed that using both glucose and crude glycerol led to improved RL production with *P. aeruginosa* compared to glucose or crude glycerol alone. Glucose (1% w/v) and glycerol (1% w/v) produced an RL titer of 1.36 g/L and 1.12 g/L, respectively. Using both glucose and glycerol (1% w/v each) produced a RL titer of 2.5 g/L. This is at least partly due to the increased quantity of carbon source fed and possibly also due to lower carbon substrate inhibition when using mixed carbon sources, as high concentrations of glucose or glycerol alone led to reduced RL production, with 2% w/v of a single carbon source producing only 0.33 and 1.02 g/L for glucose and glycerol, respectively. This demonstrates the importance of proper scoping in media formulation studies for RL production, showing the importance of considering the impact of mixed substrates.

Mixed carbon source utilization can also improve RL production in the metabolically engineered *P. putida* KT2440 from Pang et al. (2024) discussed in the “Novel rhamnolipid producers” section. The comparison was made with using glucose as the sole carbon source, where RL titers of approximately 1.2 to 1.6 g/L were achieved using 0.5 to 2% glucose. Meanwhile, using glucose and glycerol at 1% each resulted in an RL titer of 2.67 g/L. However, further increasing glycerol to 2% resulted in RL titer reduction to 2.14 g/L. As mentioned in the “Novel rhamnolipid producers” section, fed-batch fermentation produced an RL titer of 19.77 g/L and a productivity of 0.210 mg/L/h. However, it should be noted that glucose was only used in the inoculum with the base media and feeding solution containing only glycerol (Pang et al. 2024). Additionally, the higher amount of glycerol required may incentivize the substitutional use of crude glycerol generated as a biodiesel production by-product as a cheaper and more sustainable substrate. Such a route of using alternative feedstocks could also incentivize the integration of RL production into a biorefining scheme/concept, where RL production represents one pathway within many for the valorization of the fractions within complex renewable feedstocks.

#### Media optimization

As aforementioned, the selection of specific substrates alone is often not enough to maximize RL production; thus, media formulation should be combined with media optimization, especially with respect to major carbon and nitrogen sources. An exemplary study on media improvement for enhanced RL production is provided by Liu et al. (2023a, b). Using *P. aeruginosa* D1, improvements were made with respect to media formulation (carbon source selection and treatment) as well as media optimization (with respect to the carbon source). Future studies should aim not only to emulate the comprehensiveness of these works but also to go beyond the carbon source to also include other substrates, utilizing the multivariate surface response methodology discussed. As part of their development of a fermentation-foam fractionation coupling system (discussed in more detail in the “Fermentation process design and optimization” section), different carbon sources were compared, including glucose, glycerol, lauric acid, commercial edible oil, and waste frying oil. Glucose resulted in the lowest RL titer at 4.80 g/L, while commercial edible oil and waste frying oil produced approximately 12.04 and 11.38 g/L, respectively. Again, this demonstrates the preference for fatty acid–related carbon sources for RL production. The underlying reason for this is due to the lipid carbon source being catabolized via the β-oxidation pathway, which is also the main direct supplier of lipid precursors for RL synthesis (Abdel-Mawgoud et al. 2014). Liu et al. (2023a, b) attributed that the slight difference between edible oil and waste frying oil was due to stearic acid and non-metabolizable fatty acid polymers in the waste frying oil. As such, waste frying oil was selected for further use, due to cost and sustainability incentives. The concentration of oil in the media was then optimized between 1 and 4% w/v. Due to reduced oxygen mass transfer from too much oil, 2% w/v was selected, despite similar RL titers at around 11 g/L between 2 and 4% w/v oil loading. Finally, a range of emulsifiers were tested to improve the availability of the oil during fermentation, including Tween 20, CTAB, SDS, CAB-35, and RL. It was found that adding emulsifiers to the media greatly improved RL titer, up to approximately 24 g/L when using Tween 20 or RL as emulsifiers.

Liu et al. (2023a, b) only optimized for their carbon source concentration. However, to more holistically optimize multiple aspects of the media, such as the concentration of nitrogen, phosphorus, and other substrates, multivariate optimization via surface response methodology, or similar, is recommended. Zhu et al. (2024), as already mentioned in the “Novel rhamnolipid producers” section, conducted multivariate optimization via a central composite design. The authors chose to use a central composite design for substrate selection for both carbon sources (sodium citrate, glucose, beef extract) and nitrogen sources (urea, ammonium sulfate, peptone). This is unusual compared to studies in the wider fermentation central composite design literature, which typically use different levels of a particular parameter (e.g., substrate concentration) for their experimental design space. Lastly, the central composite design was also conducted with respect to the carbon-to-nitrogen (C/N) ratio. Based on the surface response, glucose and ammonium sulfate were the best of the substrates tested, and the optimal carbon-to-nitrogen ratio was 16:1. This 1.06 g/L RL produced was 4.4 times higher than the 0.24 g/L in the initial fermentation. However, the optimization in this study was quite unorthodox, and the RL production compares unfavorably to other single variable studies already discussed. Another example of an unusual set of parameters being used for optimization is Raza et al. (2020), who used Taguchi multi-objective optimization with respect to incubation time, but also substrate types and fermentation setups as their variables. This resulted in the selection of soybean waste frying oil, incubation time of 7 days, and setup III (glucose for the growth phase and waste frying oil for RL production). This did result in an increase in RL yield to 4.1 g/L. Nevertheless, the selection of qualitative parameters needs to be paired with quantitative optimization. For example, much more benefit can be gained by selecting an important variable, e.g., carbon source, then using surface response methodology to optimize the concentration.

Fortunately, more quantitative examples of multivariate optimization are present within the literature, and future works should take these studies as an example. Alcalde et al. (2024) conducted a Box–Behnken design surface response methodology with respect to the C/N ratio using sodium nitrate, carbon-to-phosphorus (C/P) ratio using potassium phosphate, calcium chloride concentration, and iron sulfate concentration. The optimal point for the surface response was at a C/N ratio of 21.172, a C/P ratio of 16.279, 0.046 g/l calcium chloride, and 0.003 g/L iron sulfate. Importantly, this optimal point was also experimentally validated, with an RL titer of 35.124 g/L, slightly higher than the predicted value of 34.027 g/L. This RL titer was 23.35% higher compared to the central point of the design. The higher carbon availability (higher C/N and C/P ratios) leading to higher RL titer is not a surprising trend. However, the optimal point was towards the edge of the design space, suggesting that an expanded design space, such as higher values of C/N and C/P ratios used, could lead to further improvements to RL production. Additional insight into the relationships between the factors could perhaps been obtained if a five-factor design was conducted, replacing the ratios respect with actual concentrations of carbon, nitrogen, and phosphorus sources. Nevertheless, this study is notable for optimizing beyond carbon and nitrogen sources, demonstrating the significant impact of proper concentration of trance elements that enhances RL production without being inhibitory.

Overall, there are good examples of studies exploring RL improvements through improving the media. Future studies should aim for more comprehensive and refined investigations, building upon the approaches discussed in this section, especially through the assessment of more holistic multivariate improvements to the media, in terms of substrate choice, substrate concentration, and emulsifying agents.

### Fermentation process design and optimization

Another important area of RL bioprocess development is the improvement and optimization of process design and operation. This can range from simply optimizing fermentation conditions and feeding regimes, to innovative setup such as in situ separation designed to reduce inhibition from RL accumulation, all of which are discussed in this section.

The simple optimization of process conditions includes Zhu et al. (2024) who in addition to media optimization discussed in the “Media formulation and optimization” section also optimized their fermentation pH and temperature to produce 1.06 g/L of RL titer (Zhu et al. 2024). Baskaran et al. (2021) also accompanied their media optimization (discussed in the “Media formulation and optimization” section) with optimized temperature (35 °C) and pH (6.5) to produce up to 2.73 g/L of RL (Baskaran et al. 2021). Arumugam and Furhana Shereen (2020), meanwhile, optimized their inoculum size (5% v/v) and pH (6.5) for simultaneous production of RL and polyhydroxyalkanoate using facultative anaerobe *Enterobacter aerogenes*. Notably, the titers were 5.381 and 4.2 g/L for RL and polyhydroxyalkanoate, respectively. This suggests that greater RL titer could be achieved by adjusting the direction of carbon flux, as conducted with producer modification discussed in the “Novel rhamnolipid producers” section. As discussed earlier in the “Novel rhamnolipid producers” section, Gong et al. (2022) investigated the impact of pH on RL production, with a focus on reduced foaming and its associated inhibitory effects. They reported a reduction in foaming behavior from 162.8 to 28.6% of the initial sample volume, between pH 8 and 4, with non-foaming conditions achieved at pH 5.5 (Gong et al. 2022).

Another important fermentation parameter that has been optimized is oxygen availability, due to its importance to cell growth, substrate consumption, and biosurfactant production (Gaur et al. 2024). Gaur et al. (2024) optimized the agitation and aeration rates for fed-batch fermentation. Central composite design was used to optimize these parameters with respect to RL titer, as well as dry cell weight, surface tension, and tensoactivity. The optimized conditions of 200 rpm agitation and 1 Lpm aeration were able to achieve 29.76 g/L of RL. This was extremely close to the value predicted by the surface response of 29.52 g/L. However, their optimal value does lie on the edge of the optimization design space. Another experimental point in the design at 200 rpm and 1.5 Lpm was able to obtain 30.22 g/L of RL. However, the surface response prediction for this point was lower than the optimal point (Gaur et al. 2024). Therefore, it is very likely that an improved experimental design space with higher agitation and aeration rates should be able to further increase RL production.

Beyond fermentation conditions, optimized feeding regimes have also been used to improve RL production. As discussed in the “Novel rhamnolipid producers” section, Zhou et al. (2023) investigated the link between nitric oxide and RL production, resulting in the modified KT1115Δ*paeKI*Δ*fhp*Δ*nirB* strain with improved nitric oxide accumulation for improved RL synthesis. Different feeding strategies of nitrogen, in the form of sodium nitrate, were then applied to this strain, including single, pulse, and continuous feeding. It was found that adding high amounts of nitrates early on in the fermentation resulted in greater RL titers and yields overall, with approximate RL titers of 35, 28, 25, and 13 g/L for 90 mmol/L at 0 h, 90 mmol/L after 24 h, 3 pulses of 30 mmol/L, and continuous feeding, respectively. The RL yields were also higher, with adding the nitrogen at 0 h having 16.3%, 36%, and 128% higher yields compared to 90 mmol/L after 24 h, 3 pulses of 30 mmol/L, and continuous feeding, respectively. Zhou et al. (2023) attributed this improvement in cultures where high nitrogen was added at 0 h to the nitrogen being used to achieve high growth early on, which could have changed the trend of intracellular NO concentration and RL production later in the fermentation. Later, the optimal feeding concentration of 10 mmol/L sodium nitrate was chosen, due to higher overall cell growth (Zhou et al. 2023).

Zhou et al. (2023) then explored a two-stage feeding process for cell growth and RL production, respectively, with no sodium nitrate fed during the first 48 h, followed by 10 mmol/L feeding. It should be noted that this strategy is tailored towards maximizing nitric oxide accumulation for improved RL production. Therefore, it was only beneficial for the engineered strain KT1115ΔpaeKIΔfhpΔnirB, allowing it to achieve high RL titer, productivity, and yield using this strategy, at 36.97 g/L, 0.308 g/L/h, and 0.80 g/g. However, the two-stage feeding strategy resulted in poorer RL production in the KT1115 and KT1115ΔpaeKI strains. So it will not be viable for other RL producers within the wider literature. Even for the engineered strain KT1115ΔpaeKIΔfhpΔnirB, the two-stage strategy also resulted in poorer cell growth (likely from lower nitrogen availability during the early stages of the process); thus, further improvements to this feeding approach can still be made.

Invally and Ju (2020) also present an exemplary case of fine-tuning nitrogen levels for optimal cell growth, foaming, and RL production via feeding. There were two initial growth phases: the first was designed for higher growth with higher nitrogen availability, while the second slower nitrogen addition was used to manage foaming. Importantly, unlike other studies, nitrogen depletion was not allowed to occur during the RL production phase, with continuous feeding of ammonium nitrate at a daily equivalent of 15% of the total nitrogen used in the growth phases. This allowed for long-term RL production, as well as higher RL titer and productivity at 120 g/L and 0.839 g/L/h, respectively (Invally and Ju 2020). This feeding regime addressed the aforementioned problem of prolonged nitrogen starvation on RL production (Sodagari and Ju 2020). Invally and Ju (2020) were able to demonstrate that long-duration (up to 505 h) cultivation demonstrated to be possible due to nitrogen additions as well as the viability of cultivation using repeated batch fermentation (with comparable RL productivities in the two production cycles at 0.560 and 0.523 g/L/h).

Jiang et al. (2021) used sequential fed-batch fermentation, which was similar to repeated batch fermentation using shake flasks, to achieve a very high RL production. Multiple cycles of shake flask fermentation at 64.4–112.8 RL titers were able to produce an overall RL titer equivalent to 552–665 g/L, with RL productivities of 1.28–1.54 g/L/h depending on the 2- or 3-day cycle times. This was a fivefold improvement in RL production over the fed-batch shake flask controls. The improved RL production was attributed to the high cell densities of up to 40 g/L maintained throughout the cycles as well as the presence of sodium nitrate and iron sulfate from the fresh media.

Another fermentation setup with potential for high productivity is continuous fermentation. However, the number of studies exploring continuous fermentation for RL production is limited. Recently, Diba et al. (2024) provided a great example of what could be achieved using chemostat RL production. A comprehensive assessment of the impact of carbon source type and concentrations (discussed in the “Media formulation and optimization” section), temperature, and most importantly, dilution/flow rates was conducted. Optimization of the flow rate is crucial in maintaining steady-state conditions by ensuring that the flow in and out of the reactor does not compromise the cell concentration (e.g., due to washout), minimizing inhibitor accumulation and helping achieve the maximum RL production. A critical dilution rate of 0.0234 h^−1^ and a maximum flow rate of 561 (mL/day) were calculated based on the Monod equation. Afterward, continuous fermentation experiments were conducted at 0, 30, 40, 55, 70, and 105% of the maximum flow rate. The flow rate of 240 mL per day was found to be the best, with a high RL yield of 0.92 g/g and 200 g/L of maximum RL titer. Furthermore, continuous fermentation was shown to be viable for 30 days, producing a total of 1182.8 g of RL (Diba et al. 2024). These results are orders of magnitude higher than the level of RL production when compared to previous continuous fermentation by Chen et al. (2021) at 3.574 g/L. The high potential and promising results for higher RL yield and productivity using repeated batch or continuous fermentation from the limited number of studies suggest that further exploration of these fermentation schemes, especially at larger bioreactor scales, should be a high-priority area for future research.

Beyond fermentation modes, other studies have explored novel fermentation designs and approaches. For example, several studies, such as the aforementioned Lin et al. (2024), have explored immobilization, which could improve RL production by reducing the impact of changes in the environment on the cells as well as facilitate cell recovery for reused in continuous fermentation (Liu et al. 2023a; Wang et al. 2020). Recently Lin et al. (2024) reported the use of *P. aeruginosa* immobilized in calcium alginate capsules in standard and thermo-responsive formulation. As this study was designed towards bioremediation, the RL titers are not comparable to dedicated RL production studies, with 1.37 and 1.53 g/L of RL produced by standard and thermo-responsive (containing N-isopropyl acrylamide) formulations, respectively, on diesel, representing up to 35% increase compared to free bacteria. The potential advantages of such an approach for bioremediation could include additional attachment space, as well as mitigation of long fermentation times, bacterial loss, and shear effects, some of which could be translated to improved RL production. The thermo-responsive formulation also provided additional benefits through the creation of channels for mass transfer via temperature changes, leading to greater RL production compared to the calcium alginate–only formulation (Lin et al. 2024). Zulkhifli et al. (2023) used *P. aeruginosa* USM-AR2 cells immobilized in polyvinyl alcohol alginate hydrogel beads. Improvements by 3% and 19% in RL production were reported in shake flasks and a custom-designed fluidized bed reactor, respectively. Similar to observations by Lin et al. (2024), cell immobilization led to extended fermentation times. Notably, the immobilized cells were able to be reused for a total of 1800 h and 15 cycles (Zulkhifli et al. 2023). Extending fermentation times, and thus reducing plant downtimes, could be key to the commercialization of RL. However, this aspect of RL development is often not explored within the literature. The comprehensive study by Liu et al. (2023b) discussed earlier also explored the use of cell immobilization within alginate-chitosan-alginate microcapsules. However, the immobilized cells only produced an RL titer of 11 g/L in comparison to the 22 g/L in free cells, which was hypotasized to be due to poorer mass transfer (Liu et al. 2023b). Therefore, immobilization by itself does not guarantee improved RL production, and a greater understanding of how the mass transfer can be improved is crucial. Although further development cell immobilization and recycling could be a useful tool in further improving repeated batch fermentations discussed earlier. Additionally, a better understanding of mass transfer within bioreactor will be crucial for future scale-up, as maintenance of mixing conditions and mass transfer is key to translating bench scale performance to pilot and commercial scales (as discussed later in the “Recent development in the context of scale-up and techno-economics” section).

Liu et al. (2023b), as part of their very comprehensive study, explored novel fermentation setup in the form of in situ foam separation, making use of the natural foam formation from the extracellular RL product. In situ separation is being explored within RL literature and wider biosurfactant literature more generally due to its potential to reduce product inhibition and provide a more concentrated crude product stream to ease downstream processing, among other benefits. For example, Chen et al. (2021) were able to increase their RL production from 3.574 to 4.744 g/L when coupling their continuous fermentation with in situ foam separation (Chen et al. 2021). In the case of Liu et al. (2023b), the fermentation process was coupled with foam fractionation, although in their case, a total RL production of 11.29 g/L appears to still be comparable to their use of immobilized cells without in situ separation (Liu et al. 2023b). This could indicate that the impact of mass transfer from cell immobilization was in their case greater than the potential benefits to be gained from in situ separation. Another example of in situ separation is Blesken et al. (2020) discussed earlier, who utilized genetic engineering to improve strain for in situ foam fractionation, reaching up to 0.24 g/L/h on their engineered strain in comparison to the SK4 strain at 0.17 g/L/h (Blesken et al. 2020). While many studies touted the potential benefit of in situ separation, the RL productivities of the current in situ separation literature are still quite low when compared to other setups, which suggests that the benefits of in situ separation are not yet properly captured in current RL production literature, leaving ample room for future exploration.

Beyond in situ separation of the product, in situ separation has also been used to remove inhibitors. Zhang et al. (2023) conducted fermentation coupled with membrane pervaporation to remove potentially inhibitory volatile organic compounds also generated by *P. aeruginosa*. With membrane separation, RL titer was improved by 134% (up to 3.6 g/L) using glycerol as a carbon source (Zhang et al. 2023). However, their overall RL production is low compared to other literature, likely due to low carbon loading and batch operation. The results from this study indicate the importance of addressing inhibitor accumulation during RL production, which is often overlooked by the wider literature.

Reactor setup and design can also be used to aid RL production via improved oxygen availability and reduced foaming. Bongartz et al. (2021 and 2023) have explored a number of novel aeration options. In their 2021 study, a membrane aeration module was utilized instead of the typical sparger and impeller setup. The comparison was made in batch fermentation between cross-flow vs dead-end aeration, with comparable RL titers. Dead-end aeration can potentially be more efficient in terms of oxygen conversion and was used in the later fed-batch study. However, the RL titer with fed-batch of 2.2 g/L (Bongartz et al. 2021) is still low compared to the literature. Despite advantages such as low energy requirements and low foaming, it appears that bubble-free membrane aeration is not suitable due to the high oxygen demand of RL production.

In Bongartz et al. (2023), agitation was instead conducted using a stirred membrane module for foam-free fermentation. Importantly, aeration was quantitatively assessed through the calculation of the maximum oxygen transfer rate, which was 175 mmol/L/h for the stirred membrane module; in comparison, their previous in situ membrane aeration was only at 55 mmol/L/h (Bongartz et al. 2021, 2023). The quantitative oxygen transfer rate should be a key parameter in RL production studies and operations due to the importance of oxygen, especially as it can be significantly impacted by process design, product accumulation, and reactor scale. Such quantification should be emulated by other future studies regardless of whether or not aeration is the core aspect of the investigation, as too many studies in the literature only state that their RL production was impeded by oxygen mass transfer limitation. Bongartz et al. (2023) also conducted a comparison to traditional aeration fermentation as a control. In the batch mode, RL titers of 1.17 and 1.37 g/L were achieved using *P. putida* KT2440 SK4 and *P. putida* KT2440 Δflag SK4, respectively. These represent a 3.5- to 4.5-fold improvement over the traditional aeration control and were attributed to the membrane aeration leading to no oxygen limitation due to foaming and no antifoaming agent addition which could have potentially negated their negative effects (Bongartz et al. 2023).

In both studies, computational fluid dynamics was shown to be a powerful tool for aiding the design and operation of the novel fermentation setup, by allowing for the modeling of key operating aspects such as fluid mixing, shear rates, and local oxygen concentration. Better and more widespread utilization of computational simulation tools could be beneficial in assessing multiple setups, designs, and operations of RL fermenters before experimental validation, saving process development time and costs. This would especially be useful for scale-up to pilot and commercial scale production.

## Recent developments in the context of scale-up and techno-economics

The recent developments discussed in the “Recent developments in rhamnolipid production” section should also be briefly put into the context of pilot-scale studies as well as techno-economic analysis, as it can shed light on how they can be used in future commercial RL production. Exemplar RL production performance from Table 1 includes the following:

- Invally and Ju (2020) with 30 g/L dry cell weight, 120.0 g/L RL titer, 0.839 g/L/h RL productivity, and 0.550 g/g RL yield
- Diba et al. (2024) with 16 g/L dry cell weights, 201 g/L RL titer, 1.64 g/L/h RL productivity, and 0.92 g/g RL yield
- Jiang et al. (2021) with up to ~ 40 g/L dry cell weights, 64.4–89.8 g/L RL titer (665 g/L overall production), and 1.54 g/L/h RL productivity

It is crucial for RL commercialization that high RL production can be achieved at larger scales. Unfortunately, as seen in Table 2, examples of pilot-scale production within the literature are not only limited but also show that there is a large room for improvement. An exception to this, Jiang et al. (2021) did note that they had achieved a high productivity of 0.8 g/L/h at a 2000-L scale. However, this was still lower than what was demonstrated with their repeated batch strategy.

**Table 2:** Rhamnolipid production performance in pilot-scale literature

Scale (L)	Dry cell weight (g/L)	RL titer (g/L)	RL productivity (g/L/h)	RL yield^a^ (g/g)	References
7.5	~ 10	1.521	0.0127^b^	-	Abo Elsoud et al. (2024)
2000	-	-	0.8	-	Jiang et al. (2021)
30	~ 18	39	0.43	0.23	Müller et al. (2010)
50	2.5	2.25	0.147	0.077	Reiling et al. (1986)

^a^Not including additional carbon compounds unaccounted for in media

^b^Calculated using data from the referenced literature

Overall, scaling up the innovations and insights from recent literature discussed in the “Recent developments in rhamnolipid production” section should be a key part of future RL development, where the main goal should be maintaining, or if possible enhancing, the fermentation performance with respect to RL titer, productivity, and yields, as these factors are critical to the economic viability of the process. This will involve addressing important areas such as agitation and aeration-related considerations (e.g., power to unit volume, flow regimes, shear stresses, and volumetric mass transfer coefficient) as well as addressing the viscosity of broth (especially in high RL titer scenarios) (de Mello et al. 2024). Future scale-up efforts may also involve the development and integration biological models, such as models for in vivo kinetics, with engineering simulations, such as computational fluid dynamics (Xia et al. 2021). Future efforts to develop larger and more effective RL production at bench, pilot, and industrial scale would require significant interdisciplinary undertaking in order to reach the scale and production necessary for an industrial biotechnology enterprise.

**Fig. 3:**
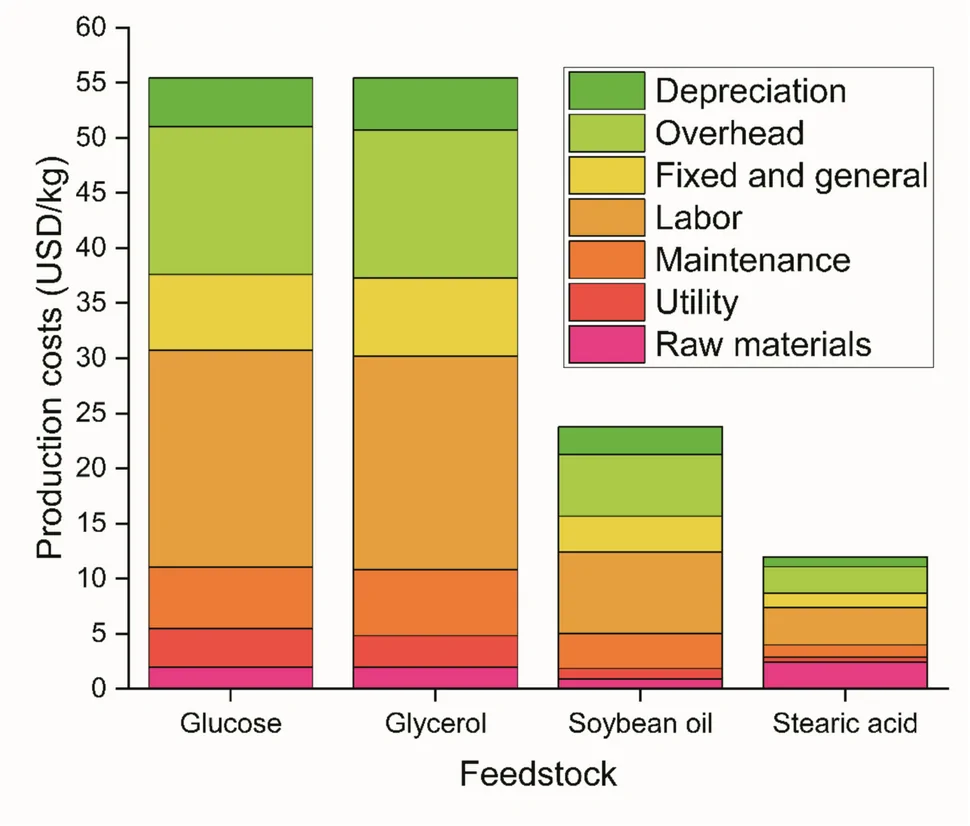
Breakdown of rhamnolipid production cost (USD/kg), produced using data from Noll et al. (2024)

Techno-economic viability will be critical for industrial RL production. However, the number and scope of techno-economic analysis of RL production in the current literature appears to still be limited. An exemplary study within this area was reported by Noll et al. (2024), who conducted both techno-economic and life cycle analysis for RL production using various feedstocks, including glucose, glycerol, soybean oil, and stearic acid. Notably, different feedstocks lead to different RL yields and productivities, which then significantly impact the important factors related to process economics, such as mass intensity, which ranged wildly from 20.95 to 111.61 kg inputs/kg di-rhamnolipid and specific energy consumption (5.05–17.84 kWh/ton raw material). This in turn led to a wide range of profit margin estimates, from 7.64 to 80.04%, the highest being for stearic acid, despite its extremely high raw material cost. For glucose, glycerol, and soybean oil, labor appears to be the highest production cost area, followed by maintenance, utility, and raw material costs. Meanwhile, raw material was nearly as high as labor in the stearic acid scenario, at 460,300 and 325,330 USD per year, respectively. Using data from Noll et al. (2024), the contributions of the various costs to the unit RL production cost can be represented, as shown in Fig. 3.

As the economic performance indicators are directly impacted by the RL fermentation performance matrices, their importance within a techno-economic analysis cannot be overstated. A comparison of performance matrices discussed in the “Recent developments in rhamnolipid production” section to the performance matrices used by techno-economic analysis within the literature (Table 3) shows that comparatively low RL yields and productivities are used as the basis of calculations in most studies/scenarios.

**Table 3:** Examples of performance values used in techno-economic studies

Scale (L)	Dry cell weight (g/L)	RL titer (g/L)	RL productivity (g/L/h)	RL yield^a^ (g/L)	Minimum selling price (USD/kg)	References
30,000	12	13	0.0887–0.514^b^	0.50–1.19	10–59	Noll et al. (2024)
-	-	-	0.0443	-	59.6	Moutinho et al. (2021)
-	-	-	-	0.037–0.043	-	Fauzi et al. (2021)

^a^Not including additional carbon compounds unaccounted for in media

^b^Calculated using data from the referenced literature

Importantly, the current literature on RL techno-economics is calculating minimum selling prices that are in many cases too high to be competitive with traditional chemical and amino acid–based surfactants, which are typically around 1.2–5 USD/kg after inflation adjustments (Bank of England 2025; Dolman et al. 2019). This is a critical point as the techno-economic analysis done within the literature assumes that the RL product can be sold at a higher price than traditional surfactants, which may not be realistic if the prices prove too high. Noll et al. (2024) reported minimum selling prices for di-rhamnolipids produced from various carbon substrates, with 58 USD/kg, 59 USD/kg, 20 USD/kg, and 10 USD/kg for glucose, glycerol, soybean oil, and stearic acid, respectively. Notably, the high productivity of 3.57 kg/t/day for stearic acid likely contributed to the low minimum selling price of the derived RL and economic viability. Moutinho et al. (2021) also reported 59.6 USD/kg for their minimum selling price, again with glycerol as the substrate. Meanwhile, Fauzi et al. (2021) assumed a selling price of 600 USD/kg, which is more in line with specialty laboratory grade chemicals and not for a commercial surfactant. The improved level of productivity and yield demonstrated in the “Recent developments in rhamnolipid production” section could be crucial in improving the market competitiveness and economic viability of RL. Therefore, future techno-economic studies should consider these innovations in order to demonstrate and quantify their potential economic impact.

## Conclusion

RL are promising biosurfactant candidates for traditional and novel applications. However, the transition from the lab to the commercial sector is still nascent. Recent developments in producer organisms, media, fermentation design, and fermentation operation could have major implications on the economic viability and market competitiveness of RL. The review has discussed in detail the promising recent studies on improved RL production, their potential implications, and areas for future studies and improvements. Future academic and industrial development of RL production and biosurfactants more generally can be aided by the discussions and recommendations provided, which should further aid the global transition towards more sustainable biochemical-based economies.

## Data Availability

No datasets were generated or analysed during the current study.
